# The relative contributions of insight and neurocognition to intrinsic motivation in schizophrenia

**DOI:** 10.1038/s41537-022-00217-z

**Published:** 2022-03-08

**Authors:** Claudio Brasso, Silvio Bellino, Paola Bozzatello, Simona Cardillo, Cristiana Montemagni, Paola Rocca

**Affiliations:** 1 Department of Neuroscience “Rita Levi Montalcini”, University of Turin, Turin, Italy; 2 Struttura Complessa Psichiatria Universitaria, Dipartimento di Neuroscienze e Salute Mentale, Azienda Ospedaliero-Universitaria “Città della Salute e della Scienza di Torino”, Turin, Italy

**Keywords:** Schizophrenia, Psychosis

## Abstract

Intrinsic motivation was described as the mental process of pursuing a task or an action because it is enjoyable or interesting in itself and was found to play a central role in the determination of the functional outcome of schizophrenia. Neurocognition is one of the most studied determinants of intrinsic motivation in clinically stable schizophrenia while little is known about the role of insight. Following this need we decided to focus on the contribution of different aspects of insight and of neurocognition to intrinsic motivation in a large sample (*n* = 176) of patients with stable schizophrenia. We performed three hierarchical linear regressions from which resulted that, among different insight aspects, the ability to correctly attribute signs and symptoms to the mental disorder made the strongest contribution to intrinsic motivation. Neurocognition, also, was significantly related to intrinsic motivation when analyzed simultaneously with insight. Moreover, even after accounting for sociodemographic and clinical variables significantly correlated with intrinsic motivation, the relationship between insight and neurocognition and intrinsic motivation remained statistically significant. These findings put the emphasis on the complex interplay between insight, neurocognition, and intrinsic motivation suggesting that interventions targeting both insight and neurocognition might possibly improve this motivational deficit in stable schizophrenia should.

## Introduction

People living with schizophrenia (SZ) often exhibit a lack of motivation that represents one of the most robust barriers to achieving functional recovery^1^. Motivation is generally defined as an internal state that initiates, directs, and maintains goal-directed behavior^2^. In particular, intrinsic motivation (IM) is described as the mental process of pursuing a task or an action because it is enjoyable or interesting in itself^3^ and was found to play a central role in learning^4,5^, in treatment response^6^, and in the determination of the functional outcome of SZ^7–13^. IM can be conceptualized as the product of a complex interplay among physiological mental processes and contextual variables^14^ and entails abilities that are often altered in SZ like reward-seeking behavior, incentive salience, and behavioral adaptations to unmet expectations and errors^15^. This conception of IM as a dynamic result of complex interactions among multiple factors poses a challenge to the development of a clear understanding of this phenomenon in SZ.

Following this need, in the last two decades, psychiatric research focused on the determinants of motivation and suggested that different clinical and cognitive variables contribute to its lack in patients living with SZ. In detail, a role was demonstrated for neurocognitive^7,9,16,17^, social cognitive^18^, and metacognitive^8,19^ deficits and for other illness-related variables, like negative symptoms^20–22^. Among other illness-related factors, Hesieh et al.^23^ found a significant relationship between insight, measured as a single total variable, and motivation for medication use.

Poor insight in SZ is prevalent across cultures and phases of illness and is associated with treatment engagement and functional outcome^24^. It includes the clinical insight, which implies the ability to recognize symptoms, needs for treatment and psychosocial consequences of the disorders and to attribute them to the illness itself^25–27^, and the cognitive insight, which involves the capacity for self-reflectiveness and resistance to excessive certainty^24,28,29^. In a broad sense, insight can be defined as the ability to have a clear, deep, and sometimes sudden understanding of a complex situation^24^ and requires the integration of multiple streams of information, including awareness of changes in internal states, external circumstances, and the views of others^30^. Multiple distinct phenomena contribute to poor insight in SZ: anomalous experiences, alterations of brain functioning, the deficit in neuro-, social and metacognition, and sociopolitical factors^24^. In particular, research on neurocognition (NC) suggests that insight is more specifically related to memory and executive functions, rather than to global intelligence^24^. However, the strength of this association is modest, suggesting that good neurocognitive abilities may be a necessary but not a sufficient condition for the development of insight^24,31^.

As previously mentioned, NC showed a significant relationship with both IM^9^ and insight^32^ and is among the main determinants of functional outcome in SZ^33^. Numerous studies documented that neurocognitive impairments are pervasive, substantial, and fundamental features of SZ. These deficits of NC are largely independent of positive symptoms, cannot be explained by the mechanism of actions of antipsychotic medications or by their side effects, are present at the time of illness onset, are relatively stable over time, and are detectable at attenuated levels in unaffected relatives of patients and in subjects considered to be at high risk for SZ^34^.

As no study directly analyzed the specific contribution of the different aspects of insight to IM, we decided to focus on this relationship. Our hypothesis was that the deficit of insight is closely linked with a lack of IM. Moreover, as both IM^9^ and insight^32^ showed a negative relationship with a neurocognitive deficit, we included NC in our analyses in order to estimate its relative contribution to the motivational deficit and its influence on the relationship between insight and IM. Furthermore, as the majority of studies focused on IM as a mediator between a single symptom or cognitive domain and functioning^7–11,16,35^ we decided to focus on the determinants of IM in SZ by taking into account insight aspects, NC, clinical, and sociodemographic variables. This kind of analysis has the potential to refine our understanding of IM deficit in SZ and thus, guide the development of therapeutic interventions with more precise targets. Consequently, the primary objective of this study was to investigate, in a large sample of patients suffering from SZ, the independent and relative contribution of various aspects of insight into illness on the levels of IM. The second objective was to clarify whether the contribution of insight could be distinguished from that of NC in explaining levels of IM. Finally, we also aimed to examine the relative contribution of insight and NC while controlling for other illness-related variables significantly related to IM.

## Results

### Characteristics of the sample and relationships with IM

Sociodemographic, clinical, NC, insight, and IM characteristics of the sample are shown in Table 1. Correlations between these variables and IM are listed in Table 2.

**Table 1:** Characteristics of the sample (*n* = 176).

*Sociodemographic variables*	
Gender (males)	100 [56.8%]
Age, years	42.07 (10.65)
Working	45 [25.6%]
Education, years	11.33 (3.59)
Stable affective relationship	46 [27.8%]
*Clinical variables*	
Age at Illness Onset, years	26.08 (9.38)
Previous hospitalizations, *N*	4.17 (5.11)
SAPS, total score	21.26 (21.84)
SANS—Avl, Score	20.20 (10.00)
SANS—ExD, Score	18.38 (13.81)
CDSS, total score	4.73 (4.65)
GAF, Score	56.40 (12.69)
*NC, insight, and IM variables*	
CVLT trials 1–5, N. of words	26.08 (9.38)
SUMD—mental disorder, Score	2.23 (1.16)
SUMD—need for treatment, Score	2.23 (1.16)
SUMD—social consequences, Score	2.71 (1.27)
SUMD—awareness of symptoms, Score	2.82 (1.13)
SUMD—attribution of symptoms, Score	3.08 (1.16)
IM, Score	8.78 (4.10)

Continuous variables are shown as means and standard deviations (SD); categorical variables as absolute number and percentage [%].

*SAPS* Scale for the Assessment of Positive Symptoms, *SANS* Scale for the Assessment of Negative Symptoms, *Avl* avolition dimension of negative symptoms, *ExD* expressive deficit dimension of negative symptoms, *CDSS* Calgary Depression Scale for Schizophrenia, *GAF* Global Assessment of Functioning, *NC* neurocognition, *IM* intrinsic motivation, *CVLT* California Verbal Learning Test, *SUMD* Scale of Unawareness of Mental Disorder, *IM* intrinsic motivation.

**Table 2:** Correlations between insight, NC, sociodemographic, and clinical variables and intrinsic motivation (*n* = 176).

Variables in the correlation	Correlation with intrinsic motivation	
	r	*p*
*Insight and NC variables*		
SUMD—mental disorder	−0.261	<0.001
SUMD—need for treatment	−0.218	0.044
SUMD—social consequences	−0.296	<0.001
SUMD—awareness of symptoms	−0.395	<0.001
SUMD—attribution of symptoms	−0.448	<0.001
CVLT trials 1–5	0.354	<0.001
*Sociodemographic and clinical variables*		
Gender (males)	−0.019	0.801
Age, years	−0.180	0.017
Education, years	0.223	0.003
Age at Illness Onset, years	−0.012	0.875
Previous hospitalizations, *N*	−0.077	0.307
SAPS total score	−0.172	0.023
SANS—ExD	−0.323	<0.001
CDSS total score	−0.085	0.263

Pearson’s correlations (r) between intrinsic motivation and insight, NC, sociodemographic, and clinical variables. Sample size is 176. Positive correlations indicate that higher scores on associated variable is related to higher intrinsic motivation.

*NC* neurocognition, *SUMD* Scale of Unawareness of Mental Disorder, *CVLT* California Verbal Learning Test, *SAPS* Scale for the Assessment of Positive Symptoms, *SANS* Scale for the Assessment of Negative Symptoms, *ExD* expressive deficit dimension of negative symptoms, *CDSS* Calgary Depression Scale for Schizophrenia.

### First objective: relative contribution of insight

Results of the first hierarchical linear regression are shown in Table 3. This regression revealed a significant effect of the global awareness of having a mental disorder (SUMD—mental disorder) on IM in Step 1, accounting for 6.8% of the variance in IM (i.e., R^2^ = 6.8%). In Step 2, adding into the model the awareness of the effect of medication on the disorder (SUMD—need for treatment) 0.1% of the variance of IM was further explained (R^2^ = 6.9%), resulting in a non-significant difference of explained variance (F of the modification—Fm— = 0.22; *p* = 0.638). Adding awareness of social consequences (SUMD—social consequences) in Step 3 explained an additional 2.9% of the variance in IM. This change was significant (Fm = 5.47; *p* = 0.021). In Step 4 the mean awareness of signs and symptoms (SUMD—awareness of symptoms) was added resulting in a significant increase of R^2^ of 7.0% (Fm = 14.42; *p* < 0.001). Finally mean attribution of signs and symptoms (SUMD—attribution of symptoms) was included in Step 5. This again led to a significant augmentation of explained variance of IM of 4.8% (ΔR^2^ = 4.8%; Fm = 10.47; *p* = 0.001). Together, the five insight measures explained about 20% of variance in IM (R^2^ = 21.6%; adjusted R^2^—adj. R^2^— = 19.3%), with attribution of signs and symptoms (SUMD—attribution of symptoms) making the largest contribution to clinical insight (standardized beta—St. B— = −0.563; *p* = 001). Moreover, with the introduction of SUMD—the attribution of symptoms into the model all the standardized beta values of the other four insight variables were no longer associated with significant *p*-values (cf. Table 3).

**Table 3:** Relative contribution of insight aspects to intrinsic motivation: first hierarchical regression analysis (*n* = 176).

Step	Variables in the model	St. β	*p*	R^2^	Adj. R^2^	ΔR^2^	Fm	*p*
1	SUMD— mental disorder	−0.261	<0.001	6.8%	6.3%	–	–	–
2	SUMD—mental disorder	−0.222	<0.001	6.9%	5.9%	0.1%	0.222	0.638
	SUMD—need for treatment	−0.052	0.638					
3	SUMD—mental disorder	−0.152	0.180	9.8%	8.2%	2.9%	5.465	0.021
	SUMD—need for treatment	0.054	0.649					
	SUMD—social consequences	−236	0.021					
4	SUMD—mental disorder	−0.050	0.658	16.8%	14.9%	7.0%	14.421	<0.001
	SUMD—need for treatment	0.106	0.357					
	SUMD—social consequences	−0.140	0.167					
	SUMD—awareness of symptoms	−0.346	<0.001					
5	SUMD—mental disorder	−0.107	0.334	21.6%	19.3%	4.8%	10.472	0.001
	SUMD—need for treatment	0.103	0.354					
	SUMD—social consequences	−0.122	0.215					
	SUMD—awareness of symptoms	0.195	0.304					
	SUMD—attribution of symptoms	−0.563	0.001					

*St.* standardized, *Adj*. adjusted, *Fm* F-value associated with the modification, *SUMD* Scale of Unawareness of Mental Disorder.

### Second objective: relative contribution of insight and NC

Results from the second hierarchical multiple linear regression are listed in Table 4. In Step 1, we found a significant effect of NC (CVLT 1–5) accounting for 12.5% of variance. In Step 2 a significant increase of R^2^ was observed (ΔR^2^ = 13.0%; Fm = 30.20; *p* < 0.001) by adding attribution of signs and symptoms (SUMD—attribution of symptoms), as unique significant predictor of IM in the first hierarchical regression. In the final model attribution of signs and symptoms made the largest contribution to IM (St. B = −0.377; *p* < 0.001), followed by NC (St. B = 0.244; *p* < 0.001). This model explained about 25% of variance in IM (R^2^ = 25.5%; adj. R^2^ = 24.6%).

**Table 4:** Relative contribution of neurocognition and insight to intrinsic motivation: second hierarchical regression analysis (*n* = 176).

Step	Variables in the model	St. β	*p*	R^2^	Adj. R^2^	ΔR^2^	Fm	*p*
1	CVLT trials 1–5	0.354	<0.001	12.5%	12.0%	–	–	–
2	CVLT trials 1–5	0.244	<0.001	25.5%	24.6%	13.0%	30.200	<0.001
	SUMD—attribution of symptoms	−0.377	<0.001					

*St.* standardized, *Adj.* adjusted, *Fm* F-value associated with the modification, *CVLT* California Verbal Learning Test, *SUMD* Scale of Unawareness of Mental Disorder.

### Third objective: relative contribution of insight, NC, sociodemographic, and clinical variables correlated to IM

Results from Step 1 of the third hierarchical linear regression (Table 5) indicated that age, education, age at illness onset, positive symptoms, and expressive deficit dimension of negative symptoms predicted 17.0% of the variance in IM. In Step 2, after adding NC (CVLT trials 1–5) into the model, an additional 4.3% of variance in IM was explained (Fm = 9.17; *p* = 0.003). Finally, in Step 3, attribution of signs and symptoms was added (SUMD—attribution of symptoms). This led to an increase in R^2^ of 8.9% (Fm = 25.05; *p* < 0.001). The final model explained about 30% of the variance in IM (R^2^ = 31.5%; adj. R^2^ = 28.6%), in which only expressive deficits, NC, and insight demonstrated a significant contribution to IM levels. In particular insight, in terms of attribution of signs and symptoms (SUMD—attribution of symptoms), made the largest unique contribution (St. B = −0.353; *p* < 0.001).

**Table 5:** Relative contribution of significantly correlated sociodemographic and clinical variables, neurocognition, and insight to intrinsic motivation: third hierarchical regression analysis (*n* = 176).

Step	Variables in the model	St. β	*p*	R^2^	Adj. R^2^	ΔR^2^	Fm	*p*
1	Age	−0.187	0.019	17.0%	14.6%	–	–	–
	Education	0.184	0.010					
	Age at Illness Onset	0.099	0.207					
	SAPS total score	−0.013	0.869					
	SANS—ExD	−0.292	<0.001					
2	Age	−0.125	0.120	21.3%	18.5%	4.3%	9.172	0.003
	Education	0.135	0.057					
	Age at Illness Onset	0.094	0.218					
	SAPS total score	0.007	0.928					
	SANS—ExD	−0.250	0.001					
	CVLT trials 1–5	0.231	0.003					
3	Age	−0.116	0.122	31.5%	28.6%	10.2%	25.047	<0.001
	Education	0.124	0.063					
	Age at Illness Onset	0.118	0.102					
	SAPS total score	0.088	0.229					
	SANS—ExD	−0.204	0.005					
	CVLT trials 1–5	0.167	0.023					
	SUMD—attribution of symptoms	−0.353	<0.001					

*St.* standardized, *Adj.* adjusted, *Fm* F-value associated with the modification, *SAPS* Scale for the Assessment of Positive Symptoms, *SANS* Scale for the Assessment of Negative Symptoms, *ExD* expressive deficit dimension of negative symptoms, *CVLT* California Verbal Learning Test, *SUMD* Scale of Unawareness of Mental Disorder.

## Discussion

The main purpose of this study was to determine the relative contribution of insight and NC to IM levels of clinically stable patients with SZ. Our results showed that the ability to correctly attribute signs and symptoms to the mental disorder (SUMD attribution of symptoms) makes the strongest contribution to IM. Moreover, we found that SUMD attribution of symptoms is the only insight aspect that maintains a significant relationship with IM when taking into account all insight variables evaluated with the SUMD. NC (CVLT trials 1–5), also, was a significant predictor of IM, however, with a weaker association in terms of lower standardized beta value as compared to SUMD attribution of symptoms. Even after accounting for sociodemographic and clinical variables significantly correlated with IM, the effect of SUMD attribution of symptoms and CVLT trials 1–5 on IM remained statistically significant indicating that both insight and NC were partly independent of the other variables added in the third hierarchical regression. Moreover, in this last regression, after the introduction of NC (CVLT trials 1–5) and insight (SUMD attribution of symptoms) variables, only the expressive deficit dimension of negative symptoms maintained a significant relationship with IM. It should be noted that the relation between the independent variables and the IM described in the regression models is a statistical association without directionality and causality as it is evaluated within a cross-sectional study.

Among insight domains evaluated with the SUMD, the ability to attribute correctly signs and symptoms to the disorder can be considered one of the most difficult to achieve, as it implies awareness of symptoms and the capacity to relate them to a mental dysfunction the patient himself is suffering from^27^. The ability to reflect upon signs and symptoms is subject to metacognitive capacities defined as the ability to think about thinking, both one’s own and the thinking of others^36^. Previous findings suggest that metacognition is a necessary building block for the development of IM in people living with SZ^8,19^ as an integrated sense of oneself and others (i.e., metacognitive abilities) likely guides goal-directed behavior by providing meaning and value to completing tasks or activities^8^. From this perspective, metacognition might be both common ground and a *trait d’union* between insight and IM. In addition, in line with the findings of Hsieh et al.^23^, a good insight would motivate people with SZ to take care of their disorder by adhering to both psychopharmacological treatments and psychosocial and rehabilitation projects, thus ameliorating the outcome in terms of real-life functioning and personal recovery. In other words, a clinical and personal recovery requires on the one hand the necessary insight to take care of oneself, on the other the motivation to implement the strategies to do so.

The contribution of NC to IM is consistent with the previous works^9,16,35^ which also found that poorer NC performance correlates with lower levels of IM. Velligan et al.^15^ proposed a putative explanation of this relationship. According to the Authors, there would be a transactional dopamine-based cognitive/motivational system that regulates volitional drive or incentive salience associated with both IM and performance in learning activities. This system would be altered in people suffering from SZ leading many patients to be indifferent to the challenges proposed by the environment they live in^15^. As for insight, also NC would be necessary to patients on the one hand to take care correctly of their disorder, for example memorizing the instructions and advice of mental health workers, on the other to be motivated to do so. Moreover, according to our results, NC contributes to the variance of IM almost independently from insight. We speculate that these two variables have independent roots converging on IM.

In this study, we did not include the avolition dimension of negative symptoms in our analysis because of its partial overlap with IM construct^20–22^; however, we found a significant contribution of the expressive deficit domain in the variance of IM, even after entering NC and insight into the regression model. Our result may be determined by the strong correlation existing between expressive deficit and avolition already reported in literature^37,38^ and also present in our data (rho = 0.67; *p* < 0.001, data not shown). In this view, we speculate that this significant contribution of expressive deficit to IM could be due to a series of concomitant correlations. Specifically, we think that the strong correlation between the expressive dimension (expressive deficit) and the experiential dimension (avolition) of negative symptoms combined with the overlap between avolition and IM constructs might lead to the statistically significant contribution of expressive deficit to IM found in our sample. Moreover, a psychopathological explanation of the contribution of the expressive deficit to IM can be added. Again, metacognition might be the connection between an independent variable, namely expressive deficit, and IM. In fact, as proposed by Jeganathan and Breakspear (2021)^39^, the metacognitive estimation of one’s causal influence over others is a prerequisite for predicting social consequences of emotive action^39^. Therefore, metacognitive deficit, such as not allowing precise inferences on the social interaction with the others, would favor blunted affect as a strategy aimed at reducing the possible damage of a manifest affectivity^39^. At the same time this deficit of metacognition, as mentioned above, would be linked to the difficulty in pursuing goal-directed action, especially those necessary for fruitful social interaction, thus resulting in a reduced IM^8^.

The main limitation is the cross-sectional nature of the study does not allow verifying if the predictors identified in this study actually precede the development of specific levels of IM. This highlights the need to further investigate whether interventions targeting patients’ insight and NC can reduce IM deficit longitudinally. Furthermore, the inclusion criteria admitted only patients that were clinically stable. This characteristic limits the generalizability of present findings to patients with more severe symptoms as during an acute phase of the illness.

Another limitation of the study is the lack of the assessment of metacognition. Further studies are needed to investigate the role of this cognitive domain in IM, focusing on its relationships with insight and NC. Moreover, the assessment of the insight with the complete version of the SUMD did not allow to assess separately the awareness and the attribution of the single principal psychopathological dimension of SZ (i.e., positive, negative, and disorganized symptoms). Therefore, the contribution of the attribution of symptoms variable to the IM cannot be analyzed in a more detailed way. Again, with regard to the appraisal of the insight, a further limitation concerns the lack of an assessment of the cognitive insight. In fact, awareness of deficits related to neurocognition and social cognition has not been investigated. This kind of insight is linked to metacognitive abilities such as the ability to reflect on one’s own cognitive abilities and to resist excessive certainty^24^. Further studies including this specific component of insight and metacognitive abilities among possible determinants of IM are necessary. NC was assessed exclusively with a verbal memory task (CVLT trials 1–5) that specifically assesses repetition verbal learning and semantic organization and provides indirect information about working memory and attention; however, to better understand the interplay between IM, insight, and NC further studies including more structured cognitive assessments are needed.

Despite these limitations, this study has some important strengths: the rather large sample size, the naturalistic design without selection bias related to randomized controlled trials, and the in-depth study of the effect of the different aspects of insight on IM levels. The findings of the present work put the emphasis on the interplay between insight, NC, and IM. Therefore, integrated psychosocial interventions targeting both insight and NC might possibly improve IM levels. For this purpose, different behavioral training-based interventions that aim to improve cognitive processes like attention, memory, executive function, social cognition, or metacognition with the goal of durability and generalization are available and validated for people living with SZ^40^. Among them computer-assisted cognitive remediation programs like CogPack and CIRCuiTS rehabilitate neurocognition and metacognitive processes related to cognitive insight^41,42^ while Social Skills Training and Social-cognitive Skills Training focus on the improvement of social cognition^43^. Cognitive insight may be targeted with specific metacognitve psychotherapies like the one proposed by Moritz et al.^44^ while clinical insight may be enhanced by psychoeducation^45^ or with recently developed psychotherapies that focus on metacognitive reflection and insight like MERIT^46^. All of these approaches have evidence of efficacy in SZ and, if properly integrated, they may improve all the cognitive abilities and the insight of patients with possible positive repercussions on IM and consequently on the functional outcomes of the disorder.

## Methods

### Participants

The study has been conducted from January 2017 and December 2020 at the Struttura Complessa Psichiatria Universitaria, Dipartimento di Neuroscienze e Salute Mentale, Azienda Ospedaliera Universitaria “Città della Salute e della Scienza di Torino”, Turin, Italy. We recruited 176 consecutive outpatients diagnosed with SZ. Inclusion criteria were: age between 18 and 65 years, willingness to participate in the study expressed through reading, understanding and signing of the appropriate informed consent, fulfillment of the formal Diagnostic and Statistical Manual of Mental Disorders 5^th^ edition (DSM-5) diagnostic criteria for SZ and clinical stability as defined below. The diagnosis of SZ was confirmed by two expert clinicians (C.B., S.C.) using the Structured Clinical Interview for DSM-5, Research Version (SCID-5-RV)^47^. Clinical stability was defined as a period of at least 3 months without hospitalization and/or treatment modifications. In addition to their medical records, all patients were considered with a stable disorder, as assessed from the reports of the patients themselves, as well as from the observations of the psychiatric staff, personnel in the psychiatric community, and relatives.

Exclusion criteria were psychiatric comorbidity with any mental disorder (DSM-5) other than SZ and a history of severe head injury (coma ≥ 48 h). The presence of psychiatric comorbidity was assessed by C.B. and S.C. using the SCID-5-RV.

Patients included in the study were evaluated using a semi-structured interview to assess demographic and clinical features. Data were collected to determine age, gender, years of education, the status of employment, marriage or an equivalent long-term relationship, age at illness onset, and the number of hospitalizations. All patients were submitted to standard care provided in community mental health centers in Italy (pharmacological treatment, clinical monitoring at least on a monthly basis, home care when required, and psychosocial and rehabilitation interventions tailored to patient’s needs).

Written informed consent was obtained from all subjects after a complete description of the study. The study was carried out in accordance with Declaration of Helsinki 1995 (as revised in Edinburgh 2000) and was approved positively by the Local Research Ethics Commitee (LREC; Protocol number: 0057625).

### Psychopathological and functioning assessment

Positive symptoms were assessed using the Scale for Assessment of Positive Symptoms (SAPS)^48^. The SAPS consists of 34 items divided into four positive symptom subscales: hallucinations, delusions, bizarre behavior, and positive formal thought disorder. The Scale for Assessment of Negative Symptoms (SANS)^49^ was employed to evaluate negative symptoms. The SANS consists of 22 items divided into five subscales (Affective Flattening or Blunting, Alogia, Avolition-Apathy, Anhedonia-Asociality, and Attention). We separated two negative symptom domains: (1) expressive deficits, which consisted of affective flattening (blunted affect) and alogia, and (2) avolition, which consisted of avolition, apathy, anhedonia, and asociality^50,51^.

Depressive symptoms were evaluated using the Calgary Depression Scale for Schizophrenia (CDSS)^52^. We used the total score of the scale where higher scores correspond to more severe depressive symptoms.

Global functioning was assessed with the Global Assessment of Functioning (GAF) scale^53^. The GAF scale is intended to be a single measure of overall impairment caused by mental factors and evaluates functioning across three domains (psychological, social functioning, and occupational/educational functioning) on a hypothetical continuum of mental health-illness. It is a single 100-point rating scale that ranges from 1, representing the hypothetically sickest, to 100, representing the hypothetically healthiest individual^54^. As proposed in previous studies^55,56^, GAF scale was employed to measure psychosocial functioning in the month before rating.

### Insight assessment

Clinical insight was assessed with the complete version of the Scale of Unawareness of Mental Disorder (SUMD)^26^, a clinician-rated standardized scale. Ratings are made after a direct patient interview. The first three items of the scale assess general unawareness of having a mental disorder (SUMD—mental disorder), the need for treatment (SUMD—need for treatment), and the social consequences of the disease (SUMD—social consequences). The scale also contains other 17 items that assess current and past unawareness and misattribution of single signs and symptoms of the disorder. Each item is rated on a 5-point Likert rating scale where higher points indicate higher unawareness/misattribution as follows: 1 = awareness/correct attribution, 2–3 partial awareness/partially correct attribution, and 4–5 unawareness/incorrect attribution. If a sign or a symptom is absent at the moment of the evaluation it is not rated. From these 17 items other two dimension of insight are calculated: awareness of the signs and symptoms (SUMD—awareness of symptoms), which is the mean of the single items scored, and attribution of the signs and symptoms (SUMD—attribution of symptoms), which is the mean of the scores of the symptoms that could be assessed because the patient is aware of them. For the purpose of the present research, we focused on current awareness and attribution of symptoms at the time of testing, namely referring to a period of time starting about one month before insight assessment.

### Assessment of NC

NC was assessed using the California Verbal Learning Test (CVLT)^57^. This test permits the assessment of multiple cognitive abilities needed to learn a listened list of words. The list consists of 16 words belonging to four different semantic categories^57^. The order of the words in the list is independent of their belonging to a specific semantic category^57^. Experimental subjects have to learn the words listened to and recall them, independently from the order they are listed. Five learning trials are performed. The word list is read, always in the same order, at the beginning of each trial. This verbal learning task involves different cognitive abilities, namely attention, short-term storing and the manipulation of the information needed to organize the words listened with a semantic criterion^58–60^. As index of the performance in the task, we employed the total number of items correctly recalled over five learning trials (CVLT trails 1–5), that is the sum of the words correctly recalled at the end of each trail.

### Assessment of IM

IM was evaluated using three items from the intrapsychic foundations subscale of the Heinrichs-Carpenter the Quality of Life Scale (QLS)^61^, namely curiosity, goal-directed motivation, and sense of purpose. QLS is a semi-structured, clinician-rated interview consisting of 21 items grouped into the following 4 domains: interpersonal relations and social network, instrumental role functioning, intrapsychic foundation, and common objects and activities. The items rated from 0 to 6, with higher scores reflecting a better QLS. This method to assess IM was proposed by Nakagami et al.^9^ and then employed in numerous subsequent empirical studies on this subject^7–9,11,17,62,63^. Higher scores reflect a greater level of motivation.

### Statistical analyses

Descriptive analyses were first conducted to characterize the sociodemographic and clinical characteristics of the participants.

Second, Pearson’s correlations were conducted between NC, measures of insight, and IM to ensure a linear relationship between these variables, needed to include them in the following regression analyses. Correlations were also applied between IM and sociodemographic and clinical variables to identify potential confounds to include in subsequent analyses. The avolition dimension of negative symptoms was not included in the correlation analysis because of its strong, even if partial, overlap with the IM construct^20–22^. Functioning measures (i.e., global functioning, employment, and relational status) also were not included in this analysis because, as proposed by previous pathway analyses^7,9,11–13,35,64,65^ we assumed that these outcome variables would be downstream of motivation in a putative pathway starting from signs, symptoms and cognitive deficits and arriving at real-world functioning throughout IM.

Three hierarchical linear regressions with IM as a dependent variable were performed to achieve the three research objectives proposed.

For our primary objective, SUMD—mental disorder was entered in the first step of the first hierarchical linear regression followed by SUMD—need for treatment, SUMD—social consequences, SUMD—awareness of symptoms, and SUMD—attribution of symptoms in the following four steps. SUMD—attribution of symptoms was entered in the last step because its scoring is contingent on SUMD—awareness of symptoms, as the attributions of signs and symptoms to the disorder require signs and symptoms awareness.

For the second research objective, CVLT trials 1–5 were entered in the first step of a second hierarchical linear regression. In Step 2 of this second regression, the insight variables found to be predictors of intrinsic motivation in the preceding regression analysis were included. CVLT trials 1–5 were entered in the first step of this regression because it was hypothesized that NC could reduce the impact of insight on IM as neurocognitive deficits were found to have strong relationships with reduced motivation. Finally, the latter hierarchical regression was repeated to address the third objective, with the addition of potential confounds identified in previous correlational analyses. Thus, sociodemographic and clinical variables that showed a significant correlation with IM were entered in the first step of this regression to remove the portion of the variance in IM that they explain and to examine whether the addition of CVLT trials 1–5 (second step) and insight (third step) allows a larger portion of the variance in IM to be accounted for.

All analyses were conducted using Statistical Package for the Social Sciences (IBM SPSS Statistics), Version 27.0, with a critical *p*-value of 0.05.

## Data Availability

The data that support the findings of this study are available on request from the corresponding author (P.R.; paola.rocca@unito.it). The data are not publicly available because they contain information that could compromise research participant privacy/consent. In particular, the anonymity guaranteed in the informed consent paperwork at the time when data were collected, data cannot be publicly shared, and are controlled by the Comitato Etico Interaziendale of the A.O.U. Città della Salute e della Scienza di Torino.

## Code availability

The code (SPSS statistics) of the analysis is available upon request to the first author (C.B.; claudio.brasso@unito.it).
